# Discrimination in the United States: Experiences of black Americans

**DOI:** 10.1111/1475-6773.13220

**Published:** 2019-10-29

**Authors:** Sara N. Bleich, Mary G. Findling, Logan S. Casey, Robert J. Blendon, John M. Benson, Gillian K. SteelFisher, Justin M. Sayde, Carolyn Miller

**Affiliations:** ^1^ Department of Health Policy and Management Harvard T.H. Chan School of Public Health Boston Massachusetts; ^2^ Research, Evaluation, and Learning Unit Robert Wood Johnson Foundation Princeton New Jersey

**Keywords:** African Americans, black Americans, discrimination, racial disparities in health and health care, racism, social determinants of health, survey research

## Abstract

**Objective:**

To examine experiences of racial discrimination among black adults in the United States, which broadly contribute to their poor health outcomes.

**Data Source and Study Design:**

Data come from a nationally representative, probability‐based telephone survey including 802 non‐Hispanic black and a comparison group of 902 non‐Hispanic white US adults, conducted January–April 2017.

**Methods:**

We calculated the percent of blacks reporting discrimination in several domains, including health care. We used logistic regression to compare the black‐white difference in odds of discrimination, and among blacks only to examine variation by socioeconomic status, gender, and neighborhood racial composition.

**Principal Findings:**

About one‐third of blacks (32 percent) reported experiencing discrimination in clinical encounters, while 22 percent avoided seeking health care for themselves or family members due to anticipated discrimination. A majority of black adults reported experiencing discrimination in employment (57 percent in obtaining equal pay/promotions; 56 percent in applying for jobs), police interactions (60 percent reported being stopped/unfairly treated by police), and hearing microaggressions (52 percent) and racial slurs (51 percent). In adjusted models, blacks had significantly higher odds than whites of reporting discrimination in every domain. Among blacks, having a college degree was associated with higher odds of experiencing overall institutional discrimination.

**Conclusions:**

The extent of reported discrimination across several areas of life suggests a broad pattern of discrimination against blacks in America, beyond isolated experiences. Black‐white disparities exist on nearly all dimensions of experiences with public and private institutions, including health care and the police. Evidence of systemic discrimination suggests a need for more active institutional interventions to address racism in policy and practice.

## 1 INTRODUCTION

From 2009 to 2016, the Obama administration established several policies aimed at reducing institutional discrimination against racial/ethnic minorities in the United States, including policies in health care, college admissions, housing, and fair lending.^1^, ^2^ However, with the Trump administration beginning to roll back these efforts in 2017, the future of reducing racial bias through federal policy and the resulting effects on minority groups have become uncertain. While recent surveys have documented large gaps between whites and blacks in their general beliefs about discrimination in the United States today,^3^, ^4^ little attention has been paid in public opinion polling to understanding disparities in personal experiences of discrimination.^5^ The objective of this study was to examine the extent of discrimination experienced by black adults in America compared to whites in response to a growing national debate about discrimination in the United States today, building on question modules from prior studies in the field.^5^, ^6^, ^7^, ^8^

Discrimination toward blacks is not just an issue that contradicts the core values of fairness and equality of opportunities in the United States. It also has very real health consequences and explains a substantial proportion of the black‐white health gap.^9^ In particular, black Americans have historically been disproportionally exposed to both institutional racism (ie, institutions, policies, and practices that perpetuate barriers to opportunities and racial disparities, such as through residential and educational segregation) and interpersonal racial discrimination (ie, directly perceived discriminatory interactions between individuals such as racial slurs or microaggressions), which are associated with major physical and mental health consequences, including mortality, hypertension, depression, anxiety, and psychological distress.^9^, ^10^, ^11^, ^12^, ^13^, ^14^ In health care settings specifically, research suggests both discrimination against racial/ethnic minorities and implicit provider biases are prevalent and negatively affect health care delivery, and patients who perceive discrimination tend to underutilize health care and forego needed medical care. However, more research on discrimination is needed using national samples.^15^, ^16^

In addition to preventing access to socioeconomic opportunities and societal resources and creating a culture that subordinates non‐white racial populations, discrimination is also directly embodied by operating as an ongoing psychosocial stressor, causing progressive wear and tear on the body's systems (known as allostatic load and overload) as it adapts to experiencing various forms of racial bias.^13^, ^17^, ^18^ Various forms of racism can be internalized,^19^ which may increase anxiety, lead to unhealthy behaviors, poor patient‐provider communication, lower levels of adherence to medical advice, increased blood pressure, or weight gain among stigmatized groups.^20^, ^21^, ^22^, ^23^ Chronic stress from everyday discrimination can lead to long‐term changes in psychological and physiologic responses,^24^ and it has contributed to persistent black‐white disparities across a range of health outcomes such as life expectancy^12^ and diet‐related disease (eg, obesity),^25^ as well as the quality of care received in the health care system.^15^, ^16^

This study brings a public health perspective to the complexity and pervasiveness of discrimination in the United States today, alongside complementary articles in this issue of *Health Services Research*. It was conducted as part of a larger nationally representative survey fielded in 2017 in response to a growing national debate about discrimination in the United States today,^1^, ^2^, ^3^, ^4^, ^5^ to understand experiences of discrimination against several different groups in America, including blacks, Latinos, Asian Americans, Native Americans, women, and LGBTQ people. Specifically, the purpose of this study was to (a) document the prevalence of racial discrimination against black adults across multiple institutional and interpersonal domains, including health care, education, employment, housing, health care, political participation, police, the criminal justice system, slurs, microaggressions, harassment, and violence; (b) to compare blacks’ experiences to whites; and (c) to examine the variation of self‐reported discrimination among black adults by gender, education, and neighborhood racial composition, as prior research suggests significant variation in blacks’ experiences of discrimination by these factors.^26^, ^27^, ^28^, ^29^, ^30^, ^31^

## 2 METHODS

### 2.1 Study design and sample

Data were obtained from an original, nationally representative, probability‐based telephone (cell and landline) survey of US adults, conducted from January 26 to April 9, 2017. The survey was jointly designed by Harvard TH Chan School of Public Health, the Robert Wood Johnson Foundation, and National Public Radio. SSRS administered the survey. Because Harvard researchers were not directly involved in data collection and de‐identified datasets were used for analysis, the study was determined to be “not human subjects research” by the Harvard TH Chan School of Public Health Office of Human Research Administration.

The full sample included 3453 US adults aged 18 years and older, and this paper examines the subsample of 802 non‐Hispanic African American or black and 902 non‐Hispanic white US adults. Throughout the paper, we use shorthand descriptors of black or white. To identify black and white adults, screening questions regarding racial and ethnic identities were asked at the beginning of the survey, which allowed interviewers to use the appropriate language about respondent's own identity in follow‐up questions. For example, this allowed questions to be read as “Did you experience [form of discrimination] because you are black?” rather than “because of your race or ethnicity?” All questions about racial/ethnic‐identity were based on respondents’ self‐identification (if respondents identified as multiracial, interviewers asked which race they identified with most).

The completion rate for this survey was 74 percent among respondents who answered initial demographic screening questions, with a 10 percent overall response rate, calculated based on the American Association for Public Opinion Research's (AAPOR) RR3 formula.^32^ Because data from this study were drawn from a probability sample and used the best available sampling and weighting practices in polling methods (eg, 68 percent of interviews were conducted by cell phone, and 32 percent were conducted via landline), they are expected to provide accurate results consistent with surveys with higher response rates,^33^, ^34^ and are therefore reliably generalizable to the broader population, within a margin of error of ± 4.1 percentage points at the 95% confidence interval. See Benson, Ben‐Porath, and Casey (2019) for a further description of the survey methodology.^35^

### 2.2 Survey instrument

The poll asked about adults’ experiences of racial discrimination. We conceptualized discrimination as differential or unfair treatment of individuals based on self‐identified race, whether by individuals (based on beliefs, words, and behavior) or social institutions (based on laws, policies, institutions, and related behavior of individuals who work in or control these laws, policies, or institution).^9^, ^12^, ^36^ We analyzed 18 questions from the survey, covering six institutional and six interpersonal areas of discrimination (full question wording in Appendix S1). Institutional areas studied were health care; employment; education; housing; political participation; and police and courts. Interpersonal areas studied were racial slurs; microaggressions (ie, negative assumptions or insensitive or offensive comments about you); racial fear; sexual harassment; being threatened or nonsexually harassed; and violence. We also examined two areas in which concerns about discrimination might prevent adults from taking needed action: seeking health services and protection from the police. We examined discrimination in domains previously demonstrated to be associated with health, as well as some that were not, in order to capture a wide range of possible discriminatory experiences across adults’ lives. We also examined general beliefs about the existence of discrimination against one's own racial group (blacks or whites) in America today. Questions about experiences were only asked among a random half sample of respondents to maximize the number of questions while limiting respondent burden. Questions were only asked of relevant subgroups (eg, college questions only asked among adults who had ever applied to college). Questions on harassment, violence, and avoiding institutions for fear of discrimination were asked about yourself or family members because of the sensitive nature of the topic and prior literature demonstrating that vicarious experiences of stress (eg, through discrimination experienced by family members) can adversely affect individuals.^37^

### 2.3 Statistical analyses

After calculating descriptive statistics, we calculated the prevalence of all blacks and whites who reported that they had ever experienced racial discrimination in each of the domains. Using pairwise t tests of differences in proportions, we made uncontrolled comparisons between the percentage of black and white adults reporting discrimination across domains. For all analyses, statistical significance was determined at *P* < .05.

We then conducted logistic regression models to assess whether reporting discrimination remained significantly associated with race after controlling for the following variables that are related to variation in experiences of discrimination: gender, age (18‐29, 30‐49, 50‐64, 65+), household income (<$25 000, $25 000‐<$50 000, $50 000‐<$75 000, $75 000+), education (less than college degree or college graduate), current health insurance status (for health care outcomes only—uninsured, Medicaid insured, non‐Medicaid insured), neighborhood racial composition (measured as whether respondents reported living in a neighborhood that is predominantly their own race or not), metropolitan status (urban, suburban, rural), and region (US Census Bureau 4‐region division: Midwest, Northeast, South, West).^38^ Among black adults only, we estimated logistic regression models to examine variation in experiences of institutional discrimination by gender, socioeconomic status (education and income), and neighborhood racial composition, while controlling for age, health insurance (for health care outcomes only), and geographic measures. To test the sensitivity of our results to model specification, we fit alternate models using different measures of discrimination, income, and education. We also tested models interacting education with age and income, but models were not predictive due to small sample sizes and were ultimately dropped from the analysis. To test characteristics associated with experiencing greater amounts of discrimination across domains, we ran an ordinal logistic regression model to estimate factors associated with experiencing between 0 and 7 institutional types of discrimination among black adults only (questions were asked among a half sample of respondents for each type of institutional discrimination).

To compensate for known biases in telephone surveys (eg, nonresponse bias) and variations in probability of selection within and across households, sample data were weighted by household size and composition, cell phone/landline use, and demographics (gender, age, education, race/ethnicity, and Census region) to reflect the true population distribution of black and white adults in the country. Other techniques, including random‐digit dialing, replicate subsamples, and random selection of a respondent within a household, were used to ensure that the sample is representative. All analyses were conducted using Stata version 15.0 (StataCorp) and all tests accounted for the variance introduced by weighted data.

## 3 RESULTS

Weighted characteristics of blacks and whites in this study sample are presented in Table 1. Blacks differed from whites on almost every demographic measure. Compared to whites, blacks were younger, less likely to have a college degree (22 vs 34 percent, *P* < .01), and more likely to live in lower‐income households (less than $25 000 per year) (41 vs 23 percent, *P* < .01). Blacks were also more likely to have Medicaid as their source of primary health insurance compared to whites (16 vs 6 percent, *P* < .01), less likely to live in a neighborhood that was predominantly their own race (35 vs 67 percent, *P* < .01), and more likely to live in the southern United States (56 vs 35 percent, *P* < .01).

**Table 1:** Characteristics of the study sample, by race^a^

	Blacks (N = 802)^b^	Whites (N = 902)^b^	*P*‐Value for Difference^c^
Percent of respondents^d^			
Gender			
Male	46	48	.51
Female	54	52	.51
Age			
18‐29 y	26	18	<.01*
30‐49 y	33	30	.26
50‐64 y	26	29	.27
65 + y	15	23	<.01*
Education			
No college degree^e^	78	66	<.01*
College degree or more	22	34	<.01*
Household income			
<$25 000	41	23	<.01*
$25 000‐<$50 000	21	22	.89
$50 000‐<$75 000	14	11	.22
$75 000+	15	35	<.01*
Don't know/refused	9	9	.84
Health insurance current status			
Uninsured	11	9	.31
Insured, Medicaid primary source	16	6	<.01*
Insured, non‐Medicaid primary source	71	84	<.01*
Living in a neighborhood that is predominantly own race^f^	35	67	<.01*
Area of residence			
Urban	32	17	<.01*
Suburban	52	53	.87
Rural	12	25	<.01*
Don't know/refused	3	5	.11
US region of residence^g^			
Northeast	17	18	.54
Midwest	16	25	<.01*
South	56	35	<.01*
West	8	18	<.01*
Don't know/refused	3	4	.22

a Non‐Hispanic black and non‐Hispanic white adults age 18+.

b The sample size shown reflects the total number of respondents in each category.

c *P*‐value for difference is based on t tests.

d Percent of US population estimated with survey weights to adjust for unequal probability of sampling; may not add up to 100% due to rounding.

e Includes those with some college experience (including business, technical, or vocational school after high school) but no college degree, as well as those with a high school degree or GED certificate or less.

f Question asked as: “People often describe some neighborhoods or areas as predominantly one group or another, such as a predominantly black or white neighborhood. Would you say that the area where you live is predominantly [respondent's own race], or not?”.

g Regions defined by US Census Bureau 4‐region definition.

* Statistically significant difference between blacks and whites at *P* < .05.

Table 2 shows unadjusted estimates of blacks and whites reporting personal discrimination because of their race across institutional domains and interpersonal domains, as well as actions based on concerns about discrimination and perceptions of general discrimination against blacks/whites in the United States today. Overall, 92 percent of blacks reported that “generally speaking, [they] believe discrimination against blacks exists in America today,” compared to 55 percent of whites reporting they believe discrimination exists against whites (*P* < .01). In the context of personally experiencing discrimination, a majority of black adults in the United States reported that they have experienced discrimination in employment—both in obtaining equal pay or promotions (57 percent) and applying for jobs (56 percent), while 60 percent reported that they or a family member have been unfairly stopped or treated by the police because they are black and 50 percent reported discrimination in police interactions. Concerns about discrimination also prevented some black adults from taking action to protect themselves: 31 percent reported that they have avoided calling the police because of concerns of discrimination, and 22 percent reported that they have avoided going to the doctor or seeking health care for themselves or family members because of concerns of discrimination or poor treatment. A majority of black adults also reported being the targets of interpersonal discrimination, as 52 percent reported hearing microaggressions and 51 percent reported hearing racial slurs. In uncontrolled comparisons, blacks were significantly more likely than whites to report experiencing discrimination in all domains, and less than one‐quarter of whites reported personal discrimination in any single domain.

**Table 2:** Unadjusted differences between black and white adults in reporting discrimination because of race^a^

	Subject of discrimination^b^	N	Black percent^c^	White percent^c^	*P*‐Value for Difference^d^
*Belief in overall discrimination*					
General belief that discrimination against [your race] exists today in the United States^e^	All adults	1704	92	55	<.01*
*Personal experiences of institutional discrimination*					
Employment					
Being paid equally or considered for promotions^f^	You	781	57	13	<.01*
Applying for jobs^g^	You	789	56	19	<.01*
Education					
Applying to or while attending college^h^	You	693	36	11	<.01*
Health care					
Going to a doctor or health clinic	You	897	32	5	<.01*
Housing					
Trying to rent a room/apartment or buy a house^i^	You	730	45	5	<.01*
Political participation					
Trying to vote or participate in politics	You	807	19	4	<.01*
Police and courts					
Interacting with police	You	807	50	10	<.01*
Unfairly stopped or treated by the police^j^	You or family member	807	60	6	<.01*
Unfairly treated by the courts^j^	You or family member	807	45	7	<.01*
*Personal experiences of interpersonal discrimination*					
Microaggressions^k^	You	897	52	19	<.01*
Racial slurs^k^	You	897	51	23	<.01*
Racial fear^k^	You	897	40	7	<.01*
Violence^j^	You or family member	807	42	13	<.01*
Threatened or nonsexually harassed^j^	You or family member	807	35	16	<.01*
Sexual harassment^j^	You or family member	807	19	9	<.01*
Actions based on concerns about discrimination					
Avoided doctor or health care because of concerns of discrimination/poor treatment	You or family member	897	22	3	<.01*
Avoided calling the police because of concerns of discrimination	You or family member	807	31	2	<.01*

a Non‐Hispanic black and non‐Hispanic white adults age 18+. Individual questions only asked among a randomized subsample of half of respondents within each race category. Don't know/refused responses included in the total for unadjusted estimates.

b Questions about you are personal experiences only; questions about you or family member ask if items have happened to you or a family member because you or they are [respondent's own race]. All adults asked about discrimination against [respondent's own race] in America today.

c Unadjusted percent, calculated using survey weights.

d *P*‐value for difference between unadjusted estimates using t tests.

e Question asked as “Generally speaking, do you believe there is or is not discrimination against [respondent's own race] in America today?”.

f Equal pay question only asked among respondents who have ever been employed for pay.

g Jobs question only asked among respondents who have ever applied for a job.

h College application/attendance was only asked among respondents who have ever applied for college or attended college for any amount of time.

i Housing question only asked among respondents who have ever tried to rent a room or apartment, or to apply for a mortgage or buy a home.

j Question wording: “Do you believe that you or someone in your family has [experienced/been _____] because you or they are [respondent's own race].”

k Question wording: “In your day‐to‐day life, have any of the following things ever happened to you, or not?” and respondent indicated they had experienced this *and* believed this happened because they are [respondent's own race]. Racial slurs = someone referred to you or a group you belong to using a slur or other negative word; microaggressions = someone made negative assumptions or insensitive or offensive comments about you; racial fear = people acted as if they were afraid of you.

* Statistically significant difference between blacks and whites at *P* < .05.

**Figure 1:**
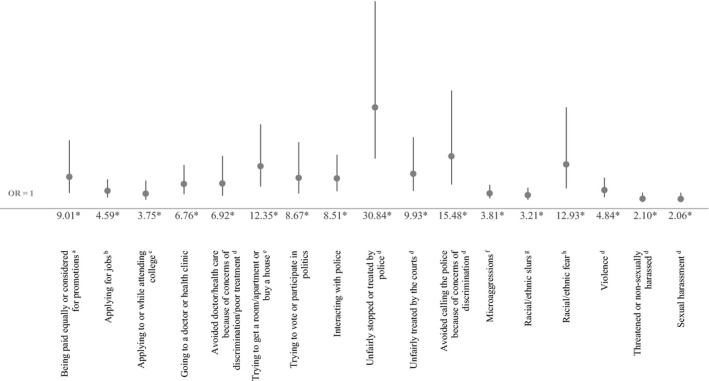
Adjusted odds of experiencing discrimination among blacks compared to whites (reference group). OR, odds ratio, with 95% confidence interval bars. Nationally representative sample of black and white adults ages 18+. * Indicates statistical significance at *P* < .05. Don't know/refused responses coded as missing. Odds ratios report the odds that blacks reported experiencing discrimination for each outcomes (whites were the reference group). These estimates control for gender, age (18‐29, 30‐49, 50‐64, 65+), education (<college vs college graduate or more), household income (<25k, 25k‐<50k, 50k‐<75k, 75k+), living in a neighborhood that is predominantly one's own race, household location (urban, suburban, rural), region (Northeast, Midwest, South, West), and for health care outcomes only, health insurance status (uninsured, Medicaid insured, non‐Medicaid insured). ^a^ Equal pay question only asked among respondents who have ever been employed for pay. ^b^ Jobs question only asked among respondents who have ever applied for a job. ^c^ College application/attendance was only asked among respondents who have ever applied for college or attended college for any amount of time. ^d^ Includes discrimination against you or a family member because you are black/white. ^e^ Housing question only asked among respondents who have ever tried to rent a room or apartment, or to apply for a mortgage or buy a home. ^f^ Microaggressions indicate that someone made negative assumptions or insensitive or offensive comments about you because you are black/white. ^g^ Racial/ethnic slurs indicate that someone referred to you or your racial group using a slur or other negative word because you are black/white. ^h^ Racial/ethnic fear indicates that people acted as if they were afraid of you because you are black/white

After we controlled for potential sociodemographic confounders in logistic regression models, all black‐white disparities in reported discrimination persisted. Figure 1 shows adjusted differences in the odds of blacks personally experiencing discrimination compared to whites. Blacks had significantly higher odds than whites in reporting discrimination in all domains, including that they or a family member were unfairly stopped or treated by the police (OR [95% CI] 30.84 [14.79, 64.34]), they have avoided calling the police because of concerns of discrimination (15.48 [6.61, 36.26]), people acted afraid of them because of their race (12.93 [5.40, 30.97]) and reporting discrimination when seeking housing (12.35 [5.97, 25.56]). Blacks also had higher odds than whites in reporting discrimination in unfair treatment by the courts against themselves or a family member (OR: 9.93 [4.58, 21.50]), obtaining equal pay/promotions (9.01 [3.95, 20.57]), police interactions (8.51 [4.54, 15.96]), when going to a doctor or health clinic (6.76 [3.57, 12.78]), and avoiding seeking health care for themselves or family members (OR: 6.92 [3.06, 15.66]).

Among blacks only, there were differences in odds of reporting discrimination by gender and socioeconomic status (education), as shown in Table 3. For socioeconomic status, having a college degree was associated with higher odds of experiencing discrimination in police interactions (2.21 [1.14, 4.30]), while household income was not associated with discrimination for any domains. For gender, black men had twice the odds of women of reporting discrimination when trying to vote or participate in politics (2.73 [1.44, 5.18]). For neighborhood racial composition, living in a predominantly black neighborhood (a measure of residential segregation) was not associated with discrimination for any domains. In the ordinal logistic regression model, having a college degree was associated with experiencing more types of discrimination (1.51 [1.06, 2.16]). This relationship was reversed among whites (data not shown). Full model results are shown in Appendix S2.

**Table 3:** Odds of reporting personal experiences of racial discrimination across institutional domains among a sample of 802 black adults in the United States

N^a^	Employment		Education	Health care		Housing	Political participation	Police and courts				Overall institutional discrimination
	Applying for jobs^b^	Equal pay/ promotions^c^	College application/ attendance^d^	Doctor or health clinic visits	Avoided doctor due to discrimination concerns	Trying to rent or buy a house^e^	Trying to vote or participate in politics	Interacting with Police	Unfairly stopped or treated by the police	Unfairly treated by the courts	Avoided calling the police due to discrimination concerns	Discrimination Across 0‐7 Domains^f^
	329	321	258	362	364	268	332	327	338	334	336	707
OR (95% CI)												
Gender												
Female	Ref	Ref	Ref	Ref	Ref	Ref	Ref	Ref	Ref	Ref	Ref	Ref
Male	1.41 (0.81, 2.45)	1.51 (0.84, 2.70)	0.97 (0.52, 1.83)	0.82 (0.48, 1.41)	1.34 (0.74, 2.46)	1.43 (0.79, 2.56)	**2.73** * (1.44, 5.18)	1.61 (0.93, 2.80)	1.56 (0.90, 2.69)	1.36 (0.80, 2.32)	1.57 (0.85, 2.87)	1.34 (0.97, 1.86)
Education												
<College	Ref	Ref	Ref	Ref	Ref	Ref	Ref	Ref	Ref	Ref	Ref	Ref
College+	1.30 (0.68, 2.51)	1.95 (0.99, 3.81)	1.14 (0.56, 2.34)	1.70 (0.86, 3.37)	1.57 (0.72, 3.44)	1.43 (0.69, 2.96)	1.11 (0.48, 2.53)	**2.21** * (1.14, 4.30)	1.20 (0.62, 2.34)	1.41 (0.73, 2.72)	0.84 (0.40, 1.75)	**1.51** * (1.06, 2.16)
Income												
<25k	Ref	Ref	Ref	Ref	Ref	Ref	Ref	Ref	Ref	Ref	Ref	Ref
25k‐<50k	0.96 (0.48, 1.91)	0.95 (0.47, 1.93)	0.91 (0.38, 2.18)	1.57 (0.75, 3.30)	0.61 (0.27, 1.36)	0.88 (0.36, 2.12)	1.30 (0.56, 3.01)	0.97 (0.47, 1.98)	1.68 (0.85, 3.34)	1.26 (0.66, 2.42)	0.99 (0.48, 2.04)	1.24 (0.80, 1.91)
50k‐<75k	0.66 (0.26, 1.65)	1.01 (0.43, 2.38)	0.90 (0.32, 2.47)	1.47 (0.62, 3.51)	0.92 (0.35, 2.46)	0.72 (0.30, 1.75)	0.64 (0.20, 2.07)	0.46 (0.20, 1.08)	2.20 (0.88, 5.51)	2.11 (0.87, 5.08)	0.99 (0.37, 2.62)	1.04 (0.66, 1.65)
75k+	1.18 (0.48, 2.91)	1.21 (0.49, 2.97)	1.29 (0.50, 3.31)	0.84 (0.35, 2.04)	0.53 (0.20, 1.40)	0.73 (0.28, 1.92)	1.20 (0.41, 3.45)	0.51 (0.21, 1.22)	1.96 (0.81, 4.77)	0.47 (0.19, 1.14)	0.37 (0.14, 0.97)	1.12 (0.69, 1.80)
Living in a predominantly black neighborhood												
No	Ref	Ref	Ref	Ref	Ref	Ref	Ref	Ref	Ref	Ref	Ref	Ref
Yes	1.23 (0.68, 2.20)	1.78 (0.97, 3.27)	0.74 (0.38, 1.42)	0.80 (0.46, 1.40)	0.70 (0.37, 1.36)	1.26 (0.66, 2.39)	1.36 (0.66, 2.81)	1.48 (0.82, 2.69)	1.43 (0.79, 2.59)	0.84 (0.48, 1.47)	1.05 (0.56, 1.95)	1.08 (0.77, 1.52)

Abbreviations: CI, confidence interval; OR, odds ratio.

a Individual questions only asked among a randomized half sample of respondents. Logistic regression models also control for three variables not shown: age (18‐29, 30‐49, 50‐64, 65+), area of residence (urban, suburban, rural), and US region of residence (South, Northeast, Midwest, West). Models for health care outcomes also adjust for insurance status (uninsured, Medicaid insured, non‐Medicaid insured). Don't know/refused responses coded as missing.

b Jobs question only asked among respondents who have ever applied for a job.

c Equal pay question only asked among respondents who have ever been employed for pay.

d College application/attendance was only asked among respondents who have ever applied for college or attended college for any amount of time.

e Housing question only asked among respondents who have ever tried to rent a room or apartment, or to apply for a mortgage or buy a home.

f Ordinal logistic regression model with experiencing discrimination in 0‐7 institutional domains as the outcome; individual questions only asked among a randomized half sample of respondents, so the maximum number of times a respondent could report experiencing discrimination along any institutional questions was 7.

* Significant at *P* < .05 (shown in bold font). Nationally representative sample of black adults ages 18+.

## 4 DISCUSSION

This paper provides a comprehensive, national snapshot about the experience of discrimination for blacks in America using recent data. We found that the overwhelming majority of black Americans perceive discrimination against blacks in America today. Black adults report personally experiencing widespread discrimination across social institutions and interpersonally, including in seeking health care, unfair treatment in by the police, and being targets of racial slurs or microaggressions. Blacks report experiencing racial discrimination at significantly higher levels than whites, regardless of gender, socioeconomic status, or the racial composition of their neighborhood. These results add to prior literature showing despite public perceptions to the contrary,^3^, ^4^, ^5^ black Americans continue to face significant barriers to equal treatment across public institutions, particularly with the police and health care, which negatively affects their health and safety.^7^, ^15^, ^39^, ^40^, ^41^ In health care specifically, the sizeable share of black adults reporting both discrimination in clinical encounters and avoiding health care due to anticipated discrimination add to research demonstrating discrimination is a significant barrier to accessing high‐quality, timely health services.^15^, ^16^ Despite specific antidiscrimination provisions included in the *Affordable Care Act* (the first federal law containing a broad prohibition on sex discrimination in health programs),^2^ discrimination in health care remains a major problem for black Americans, further amplifying health disparities between blacks and whites.

Surprisingly, we found little variation in experiences of discrimination among black adults by socioeconomic status, gender, and neighborhood racial composition, likely because of the high proportion of black adults who reported experiencing discrimination across domains. Discrimination experiences varied by domain, consistent with prior research showing that reporting discrimination varies by both demographic characteristics and the type of discriminatory treatment tested.^28^ In addition, living in a predominantly black neighborhood was not predictive of discrimination in any domains. This is inconsistent with prior research using census data which found that among blacks, living in a neighborhood with a higher percentage of blacks was associated with lower levels of discrimination.^26^, ^31^

Based on our data, having a higher household income and a college degree do not serve as protective factors against experiencing discrimination for black adults in the United States, and having a college degree was associated with *higher* odds of reporting discrimination in police interaction and more types of overall institutional discrimination across domains. These findings are consistent across prior studies showing that higher‐SES blacks report more discrimination than their lower‐SES counterparts.^9^, ^10^, ^16^, ^26^ This relationship is reversed among whites, where higher socioeconomic status is associated with reporting less discrimination. Among blacks, it is unclear whether the relationship between education and reported discrimination is driven by unequal exposures (eg, high‐SES blacks having greater contact with whites in integrated settings than low‐SES blacks) or differential reporting (eg, high‐SES blacks noticing and/or self‐reporting unequal treatment more than low‐SES blacks),^29^, ^42^, ^43^ and future research should seek to explore this relationship in greater depth.

Although there is a strong body of evidence establishing the need to narrow racial and ethnic disparities in health care,^44^ there is not a concensus on how to broadly end discrimination in health care, which would greatly reduce these disparities. In addition, achieving equity in areas as diverse as policing and voting are extremely complex, and there is no national consensus on how these problems should be solved. However, experts have recommended a myriad of different solutions worthy of serious consideration and study.^2^, ^23^, ^44^ Our data suggest that more action in this area is needed, as most interventions have not been rigorously evaluated for their effects on improving health outcomes or reducing racial health disparities. However, it is beyond the scope of our results to recommend specific policies to end discrimination in the United States.

These results also add to literature identifying important future areas of research.^20^, ^23^ Future studies should identify, implement, and rigorously evaluate policy and programmatic approaches to reduce discrimination against black Americans. Because many of the observed differences in discrimination between black and whites were not attributable to individual‐level demographic characteristics, other factors at the household, neighborhood, community, state, or national levels should be considered. Continued surveillance about the experiences of discrimination among a large, national sample of black Americans is important for tracking possible progress or decline in this area.

### 4.1 Limitations

Our results should be interpreted considering several limitations. This survey did not ask about the timing or severity of experiences of discrimination and relied on self‐reported measures. Because racial discrimination also prevents access to socioeconomic opportunities and societal resources, self‐reported experiences in this study are likely an underestimate of the aggregate effects of racial discrimination against blacks in the United States today. We also were not able to tease out potential interaction effects between age, income, and education, because of the small sample size. Question wording, ordering, and the amount of questions asked limited our ability to accurately assess experiences of discrimination across an even broader range of institutional and interpersonal areas, and explicitly inquiring about racial discrimination may have affected respondents’ answers. Our low response rate is a notable limitation, though evidence suggests that low response rates do not bias results if the survey sample is representative of the study population.^33^, ^34^ Recent research has shown that such surveys, when based on probability samples and weighted using US Census parameters, yield accurate estimates in most cases when compared with both objective measures and higher response surveys.^33^, ^34^, ^45^, ^46^ For instance, a recent study showed that across fourteen different demographic and personal characteristics, the average difference between government estimates from high response rate surveys and a poll with a response rate similar to this poll was three percentage points.^33^ However, it is still possible that some selection bias may remain that is related to the experiences being measured. Since respondents self‐identified as black, we were not able to examine heterogeneity within the US black population (eg, immigration status or skin color). In addition, when examining differences between whites and blacks across frequently reported outcomes, logistic regression models should be cautiously interpreted, as they may overstate differences between groups. Despite these limitations, this study design allowed us to closely examine reported experiences of racial discrimination among a large national sample of black adults in the United States across a range of public institutions and interpersonal experiences.

## 5 CONCLUSIONS

Efforts by the Trump administration to reverse Obama‐era federal policies aimed at reducing or preventing institutional racial discrimination highlight the major role race plays in US politics and policies today. Contrary to public discourse about high‐profile instances of discrimination that characterize them as isolated events, our results suggest that they are broadly ingrained across institutions and interpersonal interactions. The experience of discrimination for blacks in the United States is prevalent across many areas of their lives, including health care, and black adults do not receive treatment equal to whites, which other research shows carries severe economic, social, and health consequences. Findings of systemic self‐reported discrimination against blacks suggest that a greater focus is needed to create and implement major interventions and policies that address institutional patterns of discrimination.
