# Supplementary material for: Discrimination in the United States: Experiences of black Americans
Source: Health Serv Res. 2019 Oct 29;54(Suppl 2):1399–408. doi: 10.1111/1475-6773.13220 (PMC6864380; doi:10.1111/1475-6773.13220)
Supplement: Supplementary file 2 [file HESR-54-1399-s002.docx]

**Appendix S1. Survey Questions**

**Screening questions**

1. Have you ever applied for a job?
2. (Half sample): Have you ever been employed for pay?
3. (Half sample): Have you ever applied for college or attended college for any amount of time?
4. (Half sample): Have you ever tried to rent a room or apartment, or to apply for a mortgage or buy a home?

**General perceptions of discrimination**

1. Generally speaking, do you believe there is or is not discrimination against [respondent’s own race] in America today? Yes / No / (Volunteered response) Don’t know/Refused

**Institutional Discrimination**

1. (Ask if respondent has ever applied for a job): What about you? Do you believe you have ever personally experienced discrimination because you are [respondent’s own race] **when applying for jobs?** Yes / No / (Volunteered response) Don’t know/Refused
2. (Ask if respondent has ever been employed): What about you? Do you believe you have ever personally experienced discrimination because you are [respondent’s own race] **when it comes to being paid equally or considered for promotions**? Yes / No / (Volunteered response) Don’t know/Refused
3. What about you? Do you believe you have ever personally experienced discrimination because you are [respondent’s own race] **when interacting with police**? Yes / No / (Volunteered response) Don’t know/Refused
4. What about you? Do you believe you have ever personally experienced discrimination because you are [respondent’s own race] **when trying to vote or participate in politics**? Yes / No / (Volunteered response) Don’t know/Refused
5. What about you? Do you believe you have ever personally experienced discrimination because you are [respondent’s own race] **when going to a doctor or health clinic**? Yes / No / (Volunteered response) Don’t know/Refused
6. (Ask if respondent has ever applied to or attended college): What about you? Do you believe you have ever personally experienced discrimination because you are [respondent’s own race] **when applying to college or while at college**? Yes / No / (Volunteered response) Don’t know/Refused
7. (Ask if respondent has ever tried to rent/buy a place to live): What about you? Do you believe you have ever personally experienced discrimination because you are [respondent’s own race] **when trying to rent a room or apartment or buy a house**? Yes / No / (Volunteered response) Don’t know/Refused

**Interpersonal Discrimination (Against You Only)**

(Rotate items B and C, always ask A last): In your day-to-day life, have any of the following things ever happened to you, or not?

How about (INSERT)?

(IF RESPONDENT ASKS WHAT ‘GROUP’ MEANS, PLEASE SAY: Such as your race, ethnicity, gender (or your sexual orientation or identity).)

Yes, has happened / No, has not happened / (Volunteered response) Don’t know/Refused

1. Someone referred to you or a group you belong to using a slur or other negative word
2. Someone made negative assumptions or insensitive or offensive comments about you
3. People acted as if they were afraid of you

(Q9-Q11) (If Yes to previous question): Do you believe this happened to you because of your race or ethnicity, your gender, (your sexual orientation or gender identity,) or was it for some other reason? You can select multiple answers.

Race or ethnicity, Gender, Sexual orientation, Gender identity, Or some other reason (SPECIFY)

**Interpersonal Discrimination (Against You or Family)**

(Scramble items A-E; always ask B right after A)

Do you believe that you or someone in your family has (INSERT ITEM) because you or they are [respondent’s own race]? How about (INSERT ITEM)?

Yes / No / (Volunteered response) Don’t know/Refused

12. Experienced sexual harassment

13. Been threatened or non-sexually harassed

14. Been unfairly stopped or treated by the police

15. Been unfairly treated by the courts

16. Experienced violence

**Avoiding health care**

17. Have you ever avoided going to a doctor or seeking health care for you or others in your family out of concern that you would be discriminated against or treated poorly because you or they are [respondent’s own race]? Yes / No / (Volunteered response) Don’t know/Refused

**Avoiding police/law enforcement**

18. Have you ever avoided calling the police or other authority figures, even when in need, out of concern that you or others in your family would be discriminated against because you or they are [respondent’s own race]? Yes / No / (Volunteered response) Don’t know/Refused

**Neighborhood Racial Composition**

People often describe some neighborhoods or areas as predominantly one group or another, such as a predominantly black or white neighborhood. Would you say that the area where you live is predominantly [respondent’s own race], or not?

**Appendix S2. Table showing the odds of reporting personal experiences of racial discrimination across institutional domains among a nationally representative sample of Black adults in the U.S.**

|  | **Employment** | | **Education** | **Health Care** | | **Housing** | **Political Partici-pation** | **Police and Courts** | | | | **Overall Institutional Dis-crimination** |
| --- | --- | --- | --- | --- | --- | --- | --- | --- | --- | --- | --- | --- |
|  | **Applying for jobs ^b^** | **Equal pay/ promotions ^c^** | **College application/ attendance ^d^** | **Doctor or health clinic visits** | **Avoided doctor due to discrimination concerns** | **Trying to rent or buy a house ^h^** | **Trying to vote or participate in politics** | **Interacting with Police** | **Unfairly stopped or treated by the police** | **Unfairly treated by the courts** | **Avoided calling the police due to discrimination concerns** | **Discrimination Across 0-7 Domains ^f^** |
| N ^a^ | 329 | 321 | 258 | 362 | 364 | 268 | 332 | 327 | 338 | 334 | 336 | 707 |
| OR (95% CI) | | | | | | | | | | |  |  |
| **Gender** | |  |  |  |  |  |  |  |  |  |  |  |
| Female | Ref | Ref | Ref | Ref | Ref | Ref | Ref | Ref | Ref | Ref | Ref | Ref |
| Male | 1.41  (0.81, 2.45) | 1.51  (0.84, 2.70) | 0.97  (0.52, 1.83) | 0.82  (0.48, 1.41) | 1.34  (0.74, 2.46) | 1.43  (0.79, 2.56) | **2.73***  (1.44, 5.18) | 1.61  (0.93, 2.80) | 1.56  (0.90, 2.69) | 1.36  (0.80, 2.32) | 1.57  (0.85, 2.87) | 1.34  (0.97, 1.86) |
| **Education** |  |  |  |  |  |  |  |  |  |  |  |  |
| <College | Ref | Ref | Ref | Ref | Ref | Ref | Ref | Ref | Ref | Ref | Ref | Ref |
| College+ | 1.30  (0.68, 2.51) | 1.95  (0.99, 3.81) | 1.14  (0.56, 2.34) | 1.70  (0.86, 3.37) | 1.57  (0.72, 3.44) | 1.43  (0.69, 2.96) | 1.11  (0.48, 2.53) | **2.21***  (1.14, 4.30) | 1.20  (0.62, 2.34) | 1.41  (0.73, 2.72) | 0.84  (0.40, 1.75) | **1.51***  (1.06, 2.16) |
| **Income** |  |  |  |  |  |  |  |  |  |  |  |  |
| <25k | Ref | Ref | Ref | Ref | Ref | Ref | Ref | Ref | Ref | Ref | Ref | Ref |
| 25k-<50k | 0.96  (0.48, 1.91) | 0.95  (0.47, 1.93) | 0.91  (0.38, 2.18) | 1.57  (0.75, 3.30) | 0.61  (0.27, 1.36) | 0.88  (0.36, 2.12) | 1.30  (0.56, 3.01) | 0.97  (0.47, 1.98) | 1.68  (0.85, 3.34) | 1.26  (0.66, 2.42) | 0.99  (0.48, 2.04) | 1.24  (0.80, 1.91) |
| 50k-<75k | 0.66  (0.26, 1.65) | 1.01  (0.43, 2.38) | 0.90  (0.32, 2.47) | 1.47  (0.62, 3.51) | 0.92  (0.35, 2.46) | 0.72  (0.30, 1.75) | 0.64  (0.20, 2.07) | 0.46  (0.20, 1.08) | 2.20  (0.88, 5.51) | 2.11  (0.87, 5.08) | 0.99  (0.37, 2.62) | 1.04  (0.66, 1.65) |
| 75k+ | 1.18  (0.48, 2.91) | 1.21  (0.49, 2.97) | 1.29  (0.50, 3.31) | 0.84  (0.35, 2.04) | 0.53  (0.20, 1.40) | 0.73  (0.28, 1.92) | 1.20  (0.41, 3.45) | 0.51  (0.21, 1.22) | 1.96  (0.81, 4.77) | 0.47  (0.19, 1.14) | 0.37  (0.14, 0.97) | 1.12  (0.69, 1.80) |
| **Living in a Predominantly Black Neighborhood** | | | |  |  |  |  |  |  |  |  |  |
| No | Ref | Ref | Ref | Ref | Ref | Ref | Ref | Ref | Ref | Ref | Ref | Ref |
| Yes | 1.23  (0.68, 2.20) | 1.78  (0.97, 3.27) | 0.74  (0.38, 1.42) | 0.80  (0.46, 1.40) | 0.70  (0.37, 1.36) | 1.26  (0.66, 2.39) | 1.36  (0.66, 2.81) | 1.48  (0.82, 2.69) | 1.43  (0.79, 2.59) | 0.84  (0.48, 1.47) | 1.05  (0.56, 1.95) | 1.08  (0.77, 1.52) |
| **Covariates** |  |  |  |  |  |  |  |  |  |  |  |  |
| **Age** |  |  |  |  |  |  |  |  |  |  |  |  |
| 18-29 y | Ref | Ref | Ref | Ref | Ref | Ref | Ref | Ref | Ref | Ref | Ref | Ref |
| 30-49 y | **2.41***  (1.09, 5.37) | **4.04***  (1.84, 8.85) | 0.49  (0.21, 1.13) | 1.98  (0.87, 4.52) | 0.70  (0.31, 1.57) | 1.52  (0.53, 4.35) | 1.22  (0.45, 3.33) | 2.06  (0.97, 4.38) | 1.52  (0.72, 3.20) | 1.30  (0.63, 2.70) | 0.97  (0.45, 2.08) | 1.13  (0.72, 1.78) |
| 50-64 y | **3.04***  (1.37, 6.75) | **5.89***  (2.55, 13.59) | 0.78  (0.33, 1.81) | **2.93***  (1.29, 6.67) | 0.93  (0.40, 2.16) | **3.13***  (1.05, 9.36) | **3.20***  (1.27,8.03) | 1.57  (0.74, 3.36) | 0.93  (0.43, 2.00) | 0.72  (0.34, 1.49) | 0.61  (0.28, 1.32) | 1.26  (0.81, 1.95) |
| 65+ y | 1.35  (0.59, 3.06) | 2.11  (0.89, 4.99) | 0.45  (0.17, 1.18) | 2.21  (0.88, 5.59) | 0.81  (0.31, 2.11) | 0.80  (0.27, 2.33) | 0.56  (0.19, 1.68) | 0.58  (0.25, 1.30) | 0.34  (0.15, 0.75) | 0.46  (0.20, 1.01) | 0.14  (0.05, 0.39) | **0.62***  (0.40, 0.96) |
| **Health Insurance** | |  |  |  |  |  |  |  |  |  |  |  |
| Uninsured | -- | -- | -- | Ref | Ref | -- | -- | -- | -- | -- | -- | -- |
| Medicaid | -- | -- | -- | 0.72  (0.23, 2.20) | 1.02  (0.33, 3.02) | -- | -- | -- | -- | -- | -- | -- |
| Non-Medicaid | -- | -- | -- | 0.68  (0.27, 1.68) | 0.64  (0.25, 1.63) | -- | -- | -- | -- | -- | -- | -- |
| **Area of residence** | |  |  |  |  |  |  |  |  |  |  |  |
| Urban | Ref | Ref | Ref | Ref | Ref | Ref | Ref | Ref | Ref | Ref | Ref | Ref |
| Suburban | 1.25  (0.65, 2.40) | 1.17  (0.60, 2.30) | 1.04  (0.52, 2.08) | 0.82  (0.45, 1.48) | 0.94  (0.47, 1.88) | 0.76  (0.39, 1.46) | 0.86  (0.42, 1.78) | 0.95  (0.49, 1.84) | 1.88  (0.99, 5.37) | 0.96  (0.51, 1.82) | 0.78  (0.39, 1.57) | 1.22  (0.86, 1.71) |
| Rural | 1.38  (0.54, 3.54) | 2.35  (0.87, 6.34) | 1.18  (0.37, 3.77) | 1.38  (0.60, 3.20) | 1.15  (0.43, 3.06) | 0.90  (0.34, 2.39) | 1.67  (0.56, 5.02) | 0.97  (0.37, 2.53) | 2.62  (1.02, 6.70) | 1.39  (0.55, 3.48) | 1.03  (0.36, 2.95) | 1.21  (0.67, 2.18) |
| **Region of the Country** | |  |  |  |  |  |  |  |  |  |  |  |
| South | Ref | Ref | Ref | Ref | Ref | Ref | Ref | Ref | Ref | Ref | Ref | Ref |
| Northeast | 0.57  (0.26, 1.25) | 0.89  (0.42, 1.86) | 1.19  (0.49, 2.88) | 1.06  (0.47, 2.40) | 1.44  (0.66, 3.15) | 0.61  (0.26, 1.46) | 1.41  (0.57, 3.50) | 0.66  (0.2, 1.36) | 0.64  (0.29, 1.41) | 0.93  (0.44, 1.98) | 0.74  (0.31, 1.76) | 0.97  (0.64, 1.48) |
| Midwest | 1.51  (0.68, 3.35) | 1.48  (0.65, 3.38) | 2.37  (0.91, 6.16) | 1.70  (0.77, 3.74) | 1.61  (0.66, 3.95) | 0.56  (0.24, 1.30) | 2.51  (0.95, 4.84) | 1.67  (0.73, 3.83) | 1.19  (0.57, 2.50) | 1.60  (0.77, 3.34) | 0.80  (0.35, 1.87) | 1.57  (0.98, 2.52) |
| West | 0.53  (0.22, 1.28) | 0.50  (0.20, 1.28) | 0.97  (0.37, 2.53) | 2.06  (0.85, 4.99) | 1.17  (0.43, 3.25) | 1.36  (0.51, 3.60) | 0.46  (0.10, 2.20) | 1.59  (0.54, 4.67) | 0.92  (0.36, 2.37) | 0.90  (0.36, 2.23) | 0.60  (0.21, 1.73) | 0.95  (0.59, 1.53) |

Table notes: *Significant at p<0.05. Nationally representative sample of non-Hispanic Black adults ages 18+. OR=Odds Ratio, CI=Confidence Interval. ^a^ Individual questions only asked among a randomized half sample of respondents. Don’t know/refused responses coded as missing. ^b^ Jobs question only asked among respondents who have ever applied for a job. ^c^ Equal pay question only asked among respondents who have ever been employed for pay. ^d^ College application/attendance was only asked among respondents who have ever applied for college or attended college for any amount of time. ^e^ Housing question only asked among respondents who have ever tried to rent a room or apartment, or to apply for a mortgage or buy a home.  ^f^ Ordinal logistic regression model with experiencing discrimination in 0-7 institutional domains as the outcome; individual questions only asked among a randomized half sample of respondents, so the maximum number of times a respondent could report experiencing discrimination along any institutional questions was 7.
